# *Lactobacillus rhamnosus* NKU FL1-8 Isolated from Infant Feces Ameliorates the Alcoholic Liver Damage by Regulating the Gut Microbiota and Intestinal Barrier in C57BL/6J Mice

**DOI:** 10.3390/nu16132139

**Published:** 2024-07-04

**Authors:** Haiwei Liu, Dancai Fan, Jin Wang, Yuanyifei Wang, Ang Li, Sihao Wu, Bowei Zhang, Jingmin Liu, Shuo Wang

**Affiliations:** Tianjin Key Laboratory of Food Science and Health, School of Medicine, Nankai University, Tianjin 300350, China; haiweiliu927@163.com (H.L.); fandancai@163.com (D.F.); wangjin@nankai.edu.cn (J.W.); wangyyf163@163.com (Y.W.); la_official@163.com (A.L.); 1120210672@mail.nankai.edu.cn (S.W.); bwzhang@nankai.edu.cn (B.Z.); liujingmin@nankai.edu.cn (J.L.)

**Keywords:** *Lactobacillus rhamnosus*, alcoholic liver damage, gut microbiota, intestinal barrier, antioxidant

## Abstract

Alcoholic liver damage is caused by long-term or heavy drinking, and it may further progress into alcoholic liver diseases (ALD). Probiotic supplements have been suggested for the prevention or improvement of liver damage. This study was designed to consider the ameliorative effects of *Lactobacillus rhamnosus* NKU FL1-8 isolated from infant feces against alcoholic liver damage. The mice were gavaged with a 50% ethanol solution and treated with 10^9^ CFU of *L. rhamnosus* NKU FL1-8 suspension. The factors for liver function, oxidative stress, inflammation, gut microbiota composition, and intestinal barrier integrity were measured. The results showed that *L. rhamnosus* NKU FL1-8 could decrease the levels of aspartate aminotransferase (AST) to 61% and alanine aminotransferase (ALT) to 50% compared with ethanol given by gavage. It could inhibit the expression level of malondialdehyde (MDA), increase superoxide dismutase (SOD), glutathione (GSH) to relieve oxidative stress, and down-regulate the cytokines to decrease hepatic inflammation. After treatment, the level of triglycerides was reduced, and the expression levels of adenosine 5′-monophosphate (AMP)-activated protein kinase (AMPK) and the peroxisome proliferators-activated receptor-α (PPAR-α) pathway were up-regulated. Additionally, the 16S rRNA sequencing analysis showed that *L. rhamnosus* NKU FL1-8 increased the relative abundance of *Lactobacillus*, *Ruminococcaceae*, etc. At the same time, *L. rhamnosus* NKU FL1-8 could significantly reduce lipopolysaccharides (LPS) and enhance intestinal tight junction proteins. These results demonstrated that *L. rhamnosus* NKU FL1-8 could reduce the level of oxidative stress, fat accumulation, and liver inflammation caused by alcohol in the host. The underlying mechanism could be that *L. rhamnosus* NKU FL1-8 inhibits LPS by regulating the gut microbiota and repairing the intestinal barrier. Thereby, these findings support *L. rhamnosus* NKU FL1-8 as a potential functional food for the relief of ALD.

## 1. Introduction

In recent years, liver diseases, especially those caused by alcohol abuse, have been found to contribute substantially to mortality and morbidity in the global population [1]. Binge drinking is common among young people and is an area of concern in many countries worldwide [2]. Alcoholic liver disease (ALD) is a liver abnormality and dysfunction syndrome caused by long-term drinking or alcoholism, with pathological features such as lipid metabolism disorder, inflammation, and oxidative stress [3,4]. A large number of studies have shown that excessive drinking in the progression of ALD can lead to the accumulation of acetaldehyde, which can interfere with lipid metabolism and energy production, increase the synthesis and accumulation of triglycerides in the liver, and damage the liver. At the same time, alcohol can cause metabolic disorders and increase the sensitivity of the liver to toxins, thereby stimulating the inflammatory response [5]. In addition to abstinence and liver transplantation, effective treatment strategies for ALD remain to be further explored.

According to some data, more than half of alcoholics have proven intestinal barrier dysfunction and intestinal dysbiosis [6]. This increases the possibility of translocation of products of microorganisms, but also of live bacteria, into the systemic circulation, which will reach the liver. This phenomenon increases the possibility of ALD [6]. Increased permeability of the intestinal barrier, or the so-called “leaking gut”, is caused by the effect of alcohol on the tight junctions that are connected to the intestinal epithelial cells [7]. Recent studies have shown that acute alcoholism can lead to mucosal injury, quantitative and qualitative changes in the composition of the intestinal microbiota (ecological dysbiosis), and increased intestinal permeability, leading to the translocation of endotoxin (LPS) and other bacterial products into the portal vein blood stream. This altered intestinal barrier function will promote the release of LPS into the blood and then affect liver damage. In the liver, bacterial products that stimulate the release of pro-inflammatory mediums, such as reactive oxygen species (ROS), and active factors, such as tumor necrosis factor-α (TNF-α) or interleukin-1β (IL-1β), cause liver inflammatory infiltration and adipose accumulation [8]. Therefore, enhancing intestinal barrier function in ALD patients is likely to be an effective intervention based on the gut–liver axis to alleviate liver damage. Probiotics have many beneficial effects on intestinal function, including promoting intestinal development and mucosal immunity, reducing intestinal oxidative stress, alleviating diarrhea, and improving or maintaining intestinal barrier function [9]. The therapeutic potential of probiotics has been reported in animal models of ALD, where *Lactobacillus rhamnosus* CCFM1107 prevents ALD by restoring gut microbiota and reducing oxidative stress [10]. A clinical study showed that supplementation with *Lactiplantibacillus plantarum* 8PA3 and *Bifidobacterium bifidum* restored gut microbiota and improved liver enzyme levels in patients with ALD [11]. *Lactobacillus fermentum* LA12 and *Lactiplantibacillus plantarum* LC27 can attenuate alcohol-induced liver injury [12,13]. Although probiotics have some beneficial effects on intestinal function, including improved diarrhea and prolonged remission in ulcerative colitis, the exact mechanisms by which probiotics attenuate alcohol-induced liver damage remain to be elucidated.

Therefore, we screened *L. rhamnosus* NKU FL1-8 from the feces of healthy infants and established a mouse model of alcoholic liver damage. Inflammatory factors, oxidative stress factors, lipid metabolism-related indicators, gut microbiota, and intestinal barrier function-related factors in the liver and serum of mice were measured to explore the protective effect and mechanism of *L. rhamnosus* NKU FL1-8 on liver damage.

## 2. Materials and Methods

### 2.1. Chemicals and Reagents

All the chemical reagents used in this study were of analytical grade. Alcohol was purchased from Tianjin Bohua Chemical Reagent Co., Ltd. (Tianjin, China). The medium was purchased from Haibo Biotechnology Co., Ltd. (Qingdao, China) in the Island High-Tech Industrial Park. Horseradish peroxidase (HRP)-conjugated antibody was purchased from Thermo Fisher Scientific (Waltham, MA, USA). The upper rubber premix, APS, and TEMED were determined using commercial detection kits produced by Shanghai Biyuntian Biotechnology Co., Ltd. (Shanghai, China).

### 2.2. Culture of L. rhamnosus NKU FL1-8

*L. rhamnosus* NKU FL1-8 is a Gram-positive facultative anaerobe probiotic that is acid-fast and non-sporulating. It was isolated from the feces of infants who were exclusively breastfed for 40–50 days. Preparation of *L. rhamnosus* NKU FL1-8 suspension: The strains of *L. rhamnosus* NKU FL1-8 were retrieved from −80 °C storage and inoculated into MRS liquid medium with an inoculum amount of 2%. After 18 h of culture, the strains were transferred to solid plates and then transferred again to MRS liquid medium. The resulting bacterial suspension was obtained by centrifuging a 10 mL sample at 8000× *g* for 5 min, washing the precipitate three times with sterile salt buffer solution (PBS), and resuspending it in 2 mL of PBS. *L. rhamnosus* NKU FL1-8 suspension was obtained.

### 2.3. Construction of the Developmental Tree of L. rhamnosus NKU FL1-8

The strains were incubated at 37 °C to the mid-logarithmic phase, washed several times with PBS, and collected in sufficient quantity. The genomic DNA was extracted, and the quality of the extracted DNA was detected using bacterial DNA extraction kit purchased from Beijing Solebo Technology Co., Ltd. (Beijing, China). 1492R/27F primers were used to amplify the whole 16S gene of the strain, and gene sequencing and bioinformatics analysis were performed. By comparing with the local database, the closest strains to *L. rhamnosus* at the species level were selected based on 16S rRNA sequences, and the phylogenetic tree was constructed by the MEGA 6.0 software selection NJ (neighbor-joining) method.

### 2.4. The Determination of Acid Resistant Ability

*L. rhamnosus* NKU FL1-8 was cultured to the third-generation stable stage, inoculated in 1% of the volume fraction in MRS liquid medium with pH values of 3.5 and 4.5, and incubated in a constant temperature incubator at 37 °C for anaerobic culture. The bacterial suspension at 0, 3, and 6 h was diluted to the appropriate concentration, and 100 μL of diluted *L. rhamnosus* NKU FL1-8 solution was placed on the MRS solid plate. The plate was coated by ball coating. The coated plate was cultured in a 37 °C constant-temperature incubator until colonies grew.

### 2.5. The Determination of Bile Salt Resistant Ability

*L. rhamnosus* FL1-8 train to three generations, with a volume fraction of 1%, a quantity of inoculation in bile salt concentration of 0.05%, and 0.1% of MRS liquid medium put in a 37 °C constant temperature incubator for culture. The bacterial suspension at 0 and 6 h was diluted to the appropriate concentration, and 100 μL of diluted *L. rhamnosus* NKU FL1-8 solution was placed on the MRS solid plate. The plate was coated by ball coating. The coated plate was placed in a 37 °C constant-temperature incubator for anaerobic culture until colonies grew.

### 2.6. Animal Model and Experiment Design

Twenty-four healthy male C57BL/6J mice (SPF grade, 8 weeks old) from Beijing Weitong Lihua Biological Co., Ltd. (Beijing, China) were used. They were housed in cages in the animal room at Nankai University’s Laboratory Animal Center (permission number: SYKX 2019-0001). The animals had arbitrarily chosen diet and water and followed a daily light–dark cycle of 12 h each (2023-SYDWLL-000430).

After one week of acclimation, the mice were divided into three groups, with eight mice in each group. The groups included a control group (NC), a model group (MC), and a *L. rhamnosus* NKU FL1-8 group (FL1-8). Each day at 9 a.m., the NC group received oral gavage administration of 10 mL/kg PBS, while the MC and FL1-8 groups received oral gavage administration of a 10 mL/kg ethanol solution containing 50% ethanol. At 2 p.m. every day for three weeks, the NC and MC groups received oral gavage administration of 0.2 mL PBS, while the FL1-8 group received oral gavage administration of 0.2 mL bacterial suspension containing 5 × 10^9^ CFU/mL *L. rhamnosus* NKU FL1-8. The mice were fasted for 12 h at the end of the experiment, and blood samples were collected after anesthesia and sacrificed. The serum and organs were collected and stored in the refrigerator at −80 °C for further storage. Tissues used for pathological section analysis were immersed in a 4% paraformaldehyde solution.

### 2.7. Serum and Liver Biochemical Analysis

The levels of ALT and AST were determined according to the commercial detection kits from Nanjing Jiancheng Bioengineering Institute (Nanjing, China). Serum and tissue levels of TNF-α, IL-1β, and IL-6 were measured by enzyme-linked immunosorbent assay (ELISA) kits from Shanghai Enzyme Linked Biotechnology Co., Ltd. (Shanghai, China). Liver tissues were homogenized in phosphate buffered saline (PBS), centrifuged, and the supernatant collected. Subsequently, commercial kits were used for SOD, GSH, and MDA assay kits. Total cholesterol (TC) and triglyceride (TG) were purchased from Nanjing Jiancheng BioEngineering Research Institute Co., Ltd. (Nanjing, China). All data were quantified according to the manufacturer’s instructions. Serum levels of LPS were measured by (ELISA) Jiangsu Meimian Industrial Co., Ltd. (Jiangsu, China).

### 2.8. Histopathological Examination

Paraffin embedding tissue and slice. Respectively with hematoxylin and eosin (H&E) (Beijing Solarbio Science & Technology Co., Ltd., Beijing, China) and oil red O staining on 5 micron-thick slices. Corresponding images were taken with an inverted fluorescence microscope (Olympus IX73, Olympus, Japan). Lipid droplet areas of oil red O-stained sections were analyzed using ImageJ software (1.46r). The villus length was analyzed by Slide Viewer software (2.5.0.143918). Three randomly selected regions for each sample were averaged as the value for that sample.

### 2.9. Western Blot Analysis

Liver homogenate in mice with RIPA buffer and 1% protease inhibitors mix, and at 14,000 RPM, 4 ° C for 15 min. Protein content in the supernatant was determined using a protein assay kit (BCA, Thermo Fisher Scientific, Waltham, MA, USA). The isolated proteins were detected by SDS-PAGE and transferred to PVDF membranes. After blocking with 5% skim milk, these membranes were probed with antibodies at 4 °C. Primary antibodies were purchased from Abcam (Waltham, MA, USA) and Wuhan Sanying Co., Ltd. (Wuhan, China). Anti-β-actin antibody was provided by Cell Signal Technology (Danvers, MA, USA). After washing three times with TBST, the secondary antibody detection membrane was incubated for 60 min at room temperature. The ECL chemiluminescence kit (Thermo Scientific, Carlsbad, CA, USA) was used for color development. Imaging was performed using an ultrasensitive gel imager (BIO-RAD, Hercules, CA, USA).

### 2.10. Immunofluorescence Analysis

The expression of Muc2, Zo-1 and occludin in the intestinal mucus layer was stained. The sections were deparaffinized and hydrated for 1 h, then Tris-EDTA buffer was added and cooked in a microwave oven for 3 min for antigen repair. Using sheep serum, the cells were blocked at 37 °C for half an hour and then incubated overnight at 4 °C with primary antibody. Fluorescent HRP was then added and incubated for 1 h at room temperature. Sealed sections containing DAPI were added, and finally used for microscopy.

### 2.11. Assessment of Expression Levels of Relevant Genes by RNA Extraction and q-PCR

Total RNA was extracted from the colon tissue of each mouse using a phenol-based method according to the manufacturer’s instructions (TRIzol^®^Reagent, Takara, Dalian, China). After total RNA extraction, cDNA was rapidly synthesized using SuperScript III reverse transcriptase (Invitrogen, Waltham, MA, USA). The relative mRNA expression levels in the liver, ileum, and colon were measured using the SYBR Premix Ex Taq™QPCR Kit (RR420A, Takara). Target gene expression (2^−ΔΔCt^) was normalized to endogenous β-actin expression. The primers used are listed in Table 1.

### 2.12. Gut Microbiota

The gut microbiome was analyzed by 16S rRNA gene sequencing. The procedure involves the extraction of microbial DNA and checking for gene validity using gel electrophoresis. After purification and identification, specific primers with the V3–V4 region of the 16S rRNA coding gene were used to amplify the V3–V4 domain of 16S rRNA. The Qubit 3.0 fluorometer purchased from Thermo Fisher Scientific (Waltham, MA, USA) was used for recovery and quantitative amplification, and equal amounts of fluorometers were mixed and connected to the sequencing connector. The sequencing library was constructed according to the official Illumina details, and the sequencing was completed using the PE250 model in the Hiseq2500 system from Illumina (San Diego, CA, USA). After filtering and merging the raw data, the operational taxonomic unit (OTU) results were obtained with 97% similarity to the cross-cut. To reduce the effect of low-abundance OTUs on the overall analysis, OTUs with an abundance lower than 2 were filtered out and not involved in subsequent analyses. Based on species richness information in OTUs, the Chao1 index was used to obtain an estimate of α-diversity. T test and other statistical methods were used to analyze the differences in the composition and abundance of gut microbiota in each group. α-diversity: Chao1 stands for diversity index calculated in QIIME, β-diversity: PLS-DA analysis was performed using the ropls package of the R language. Indicator species analysis: Stacked maps of species abundance were presented using the R language ggplot2 package, LEfSe software (1.0), randomforest package, pROC package, and labdsv package to screen biomarker species in each group.

### 2.13. Statistical Methods and Data Processing

Comparisons among all groups were performed by one-way analysis of variance with a post hoc Tukey’s test. When *p* < 0.05 m, the difference was statistically significant. Data processing software used in USES is SPSS (modeler 18.4). The results of the groups are the average error of the mean plus or minus. Data on the gut microbiota were analyzed with the use of the Omicsmart online platform (http://www.omicsmart.com, accessed on 15 November 2023).

## 3. Results

### 3.1. Colony Morphology and Its Characteristic Parameters

**Figure 1:**
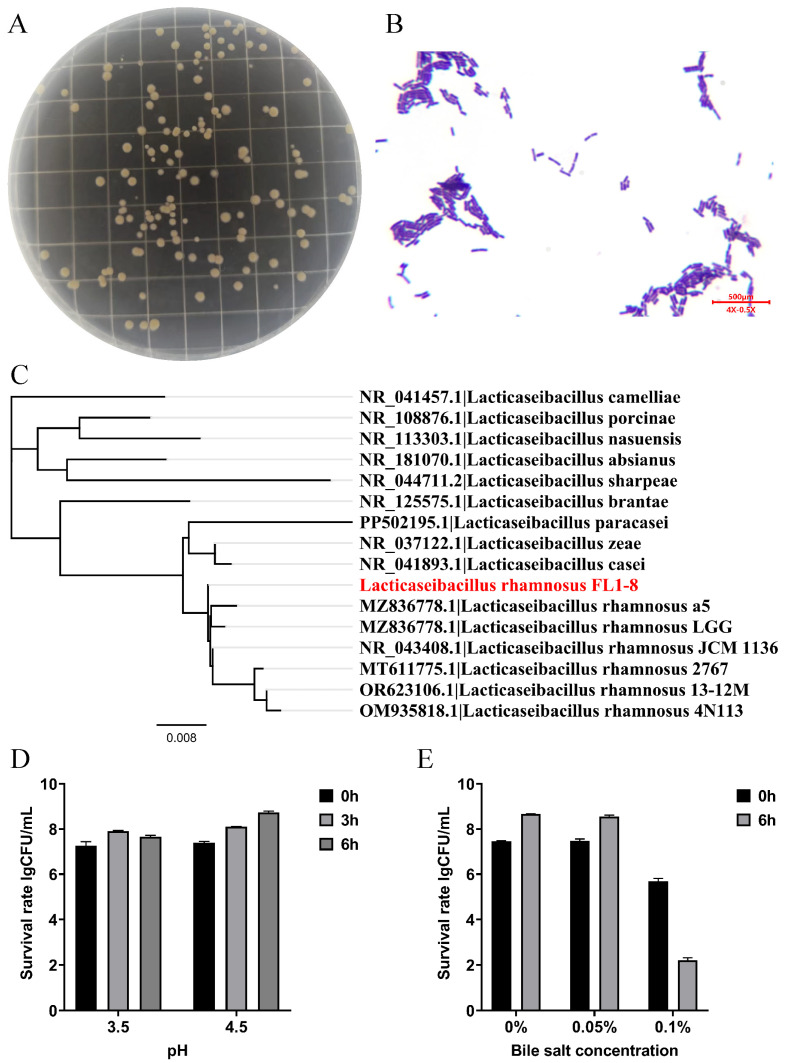
Physical properties of *L. rhamnosus* NKU FL1-8. (**A**) Colony morphology of *L. rhamnosus* NKU FL1-8; (**B**) *L. rhamnosus* microscopic form; (**C**) 16S rRNA phylogenetic tree of *L. rhamnosus* NKU FL1-8 and its closely related strains (*L. rhamnosus* NKU FL1-8 is indicated in red); (**D**) the number of viable bacteria at different pH; (**E**) the number of viable bacteria bile salt concentrations.

*L. rhamnosus* NKU FL1-8 colonies on MRS solid medium were small, smooth, and milky white, with neat and raised edges and a soft texture (Figure 1A). Gram staining of the bacteria was positive under the light microscope (Figure 1B), and the morphology of the strains had a ball-and-stick shape after primary separation or subculture. Then identify the bacterial strains and construct a phylogenetic tree. As a result, *L. rhamnosus* NKU FL1-8 was identified as *L. rhamnosus* with 99% sequence similarity to other strains of the genus *L. rhamnosus*, which was further confirmed as *L. rhamnosus* (Figure 1C). Then the characteristics of the acid and bile salt tolerance of the strain were explored. The survival rate of the strain was 109.3 ± 0.05% after 3 h of culture at pH 4.5, and the survival rate continued to rise to 118.10 ± 0.10% after 6 h of culture at pH 3.5, and the survival rate was 109.5 ± 0.01% after 3 h of culture at pH 3.5. Cultivating 6 h after survival was a 106.0 ± 0.08% drop (Figure 1D); after culturing in MRS medium containing 0.05% and 0.1% bile salts for 6 h, the survival rate reached 114.45 ± 0.08% and 38.71 ± 0.20%, respectively (Figure 1E). The results showed that the bacteria had good acid tolerance and bile salt tolerance.

### 3.2. Body Weight and Serum Indicators

**Figure 2:**
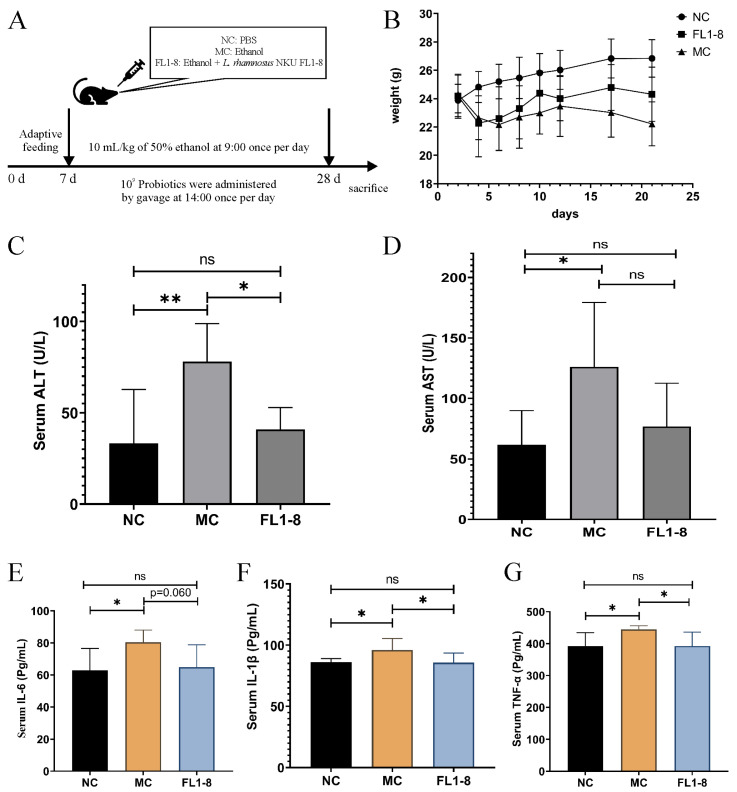
Analysis of liver function and inflammation in mice. (**A**) Animal experiment design; (**B**) body weight gain of mice; (**C**) serum ALT level; (**D**) serum AST level; (**E**) serum IL-6 level; (**F**) serum IL-1β level; (**G**) serum TNF-α level. Data are presented as mean ± standard deviation (*n* = 8); * *p* < 0.05, ** *p* < 0.01, ns: *p* > 0.05.

The experimental scheme is shown in Figure 2A. As shown in Figure 2B, the body weight of experimental mice was significantly reduced after gavage with alcohol compared with the NC group. For the NC group, the body weight of mice showed an upward trend from 0 to 21 days. For the model group, the body weight of mice decreased rapidly from day 0 to day 6, then increased from day 6 to day 12, and decreased steadily from day 12. The body weight of mice in the FL1-8 group was similar to that in the MC group from day 0 to day 4, with a sharp decrease in body weight. Then, the body weight of mice in the FL1-8 group returned to the initial body weight level from day 4 to day 10 and then remained stable. The rapid weight loss in the MC group and FL1-8 group was due to stress caused by a sudden high alcohol intake. For the MC group, after adaptation to alcohol intake (6–12 days), the body weight recovered to some extent, but the body weight was still low, while the mice treated with FL1-8 could resist the rapid weight loss induced by alcohol. ALT and AST are the most commonly used sensitive indicators. When 1% of hepatocytes are necrotic, serum ALT levels can be doubled, and ALT levels are directly proportional to the degree of liver damage [14]. Compared with the NC group, the serum levels of ALT and AST in the MC group were significantly increased (Figure 2C,D). However, the levels of ALT in the FL1-8 group were significantly lower than those in the MC group, and there was no significant difference between the NC group and *L. rhamnosus* NKU FL1-8 after the intervention with the AST levels compared with the MC group—no significant difference. These results indicate that *L. rhamnosus* NKU FL1-8 can effectively alleviate alcohol-induced liver damage in mice. The results of the levels of inflammatory factors in serum showed that the concentrations of TNF-α, IL-6, and IL-1β in the MC group were all significantly higher than those in the NC group (Figure 2E−G). The concentrations of TNF-α and IL-1β in the FL1-8 group were significantly lower than those in the MC group and were not significantly different from those in the NC group. Interestingly, the IL-6 level in the FL1-8 group was not significantly lower than that in the MC group (*p* = 0.060). This suggests that the intervention of *L. rhamnosus* can reduce the level of inflammation in serum.

### 3.3. The Pathology and Oxidative Stress Level in Hepatic

**Figure 3:**
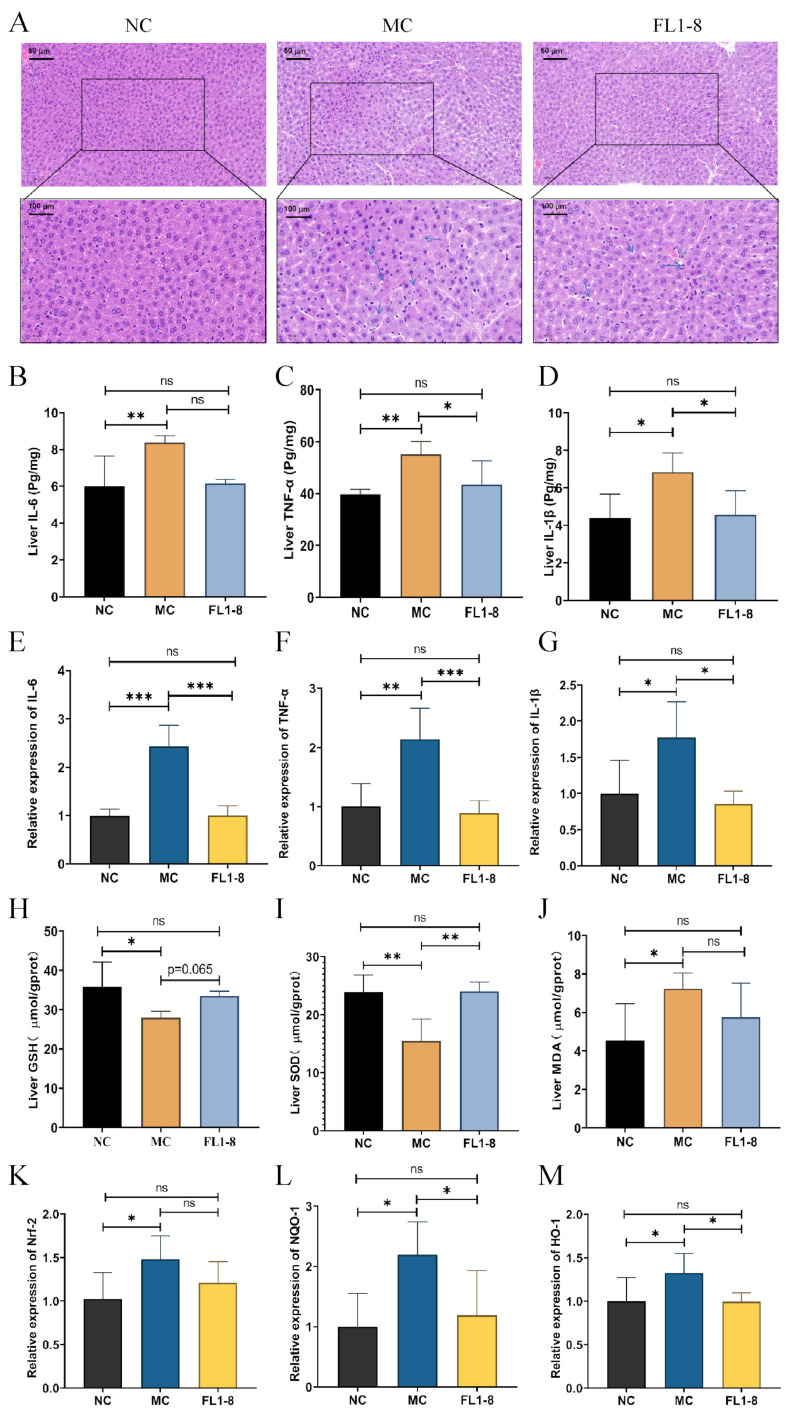
Histopathological observations and inflammatory and oxidative stress analysis of mice. (**A**) Liver H&E staining (20×) (40×). Fat vacuoles are indicated by arrows; (**B**) IL-6 protein expression in liver; (**C**) liver TNF-α protein expression level; (**D**) IL-1β protein expression in liver; (**E**) hepatic IL1-6 gene expression; (**F**) liver TNF-α gene expression level; (**G**) hepatic IL-1β gene expression; (**H**) liver GSH level; (**I**) liver SOD level; (**J**) liver MDA level; (**K**) Nrf-2 gene expression level in liver; (**L**) NQO-1 gene expression level hepatic; (**M**) HO-1 gene expression level in liver. Data are mean ± standard deviation (*n* = 8); *p < 0.05, ** *p* < 0.01, *** *p* < 0.001, ns: *p* > 0.05.

Staining was used to observe the liver damage, and Figure 3A showed that the hepatocytes in the NC group had normal structure, regular morphology, clear spacing, and no necrotic cells or inflammatory cell infiltration. In the MC group, the structure of some liver cells was found to be destroyed, showing a disordered form, with the formation of fat vacuoles, and the connection between liver cells was not tight, which was consistent with the characteristics of acute alcoholic liver damage reported in the literature [15]. Compared with the MC group, the FL1-8 group showed a more compact arrangement and fewer fat vacuoles.

In ALD cases, hepatic steatosis can lead to concomitant changes in the gene expression levels of proinflammatory factors such as TNF-α, IL-6, and IL-1β [16]. Compared with the NC group, the mRNA expression of TNF-α, IL-6, and IL-1β was significantly increased in the MC group, which was prevented in the FL1-8 group (Figure 3E–G). By measuring the protein levels of inflammatory factors, compared with the NC group, the protein expressions of TNF-α, IL-6, and IL-1β in the MC group were significantly increased, while there was no significant difference between the FL1-8 group and the NC group (Figure 3B–D). This suggests that intervention with *L. rhamnosus* NKU FL1-8 indeed reduces the expression of inflammatory factors in the liver after alcohol consumption. To investigate the regulatory effect of *L. rhamnosus* NKU FL1-8 on oxidative stress in the liver of ALD mice, the contents of GSH, MDA, and SOD in the liver were measured. The results showed that after alcohol intervention, the levels of SOD and GSH in the liver of mice were significantly decreased (Figure 3H,I), while after the intervention of *L. rhamnosus* NKU FL1-8, the decreasing trend of SOD and GSH levels was alleviated. On the other hand, we measured MDA levels in the liver and found that the MC group had a significant increase in MDA levels compared with the NC group (Figure 3J), while the FL1-8 group was not significantly different from the NC group. This is further evidence that *L. rhamnosus* NKU FL1-8 can effectively increase the reducing agents and reduce oxidation products so as to cope with the oxidative stress induced by alcohol. The inducer 3H-1, 2-dithiole-3-thione, induced the activation of the nuclear factor erythroid 2 (NF-E2)-related factor 2 (Nrf2) pathway. The significant expression of heme oxygenase 1 (HO-1) and NADPH:quinone oxidoreductase 1 (NQO1) can be significantly observed, which may be an important way to protect liver cells against oxidative damage [17]. Figure 3K–M shows that alcohol intake increased the gene expression levels of Nrf2 and HO-1 as well as NQO1 in the MC group compared with the NC group, and the gene expression levels of Nrf2, NQO1, and HO-1 in the *L. rhamnosus* NKU FL1-8 group were significantly lower than those in the MC group. Therefore, we hypothesized that *L. rhamnosus* NKU FL1-8 may, through Nrf2/HO-1, enhance the antioxidant defense system of ALD.

### 3.4. Levels of Lipid Metabolism

**Figure 4:**
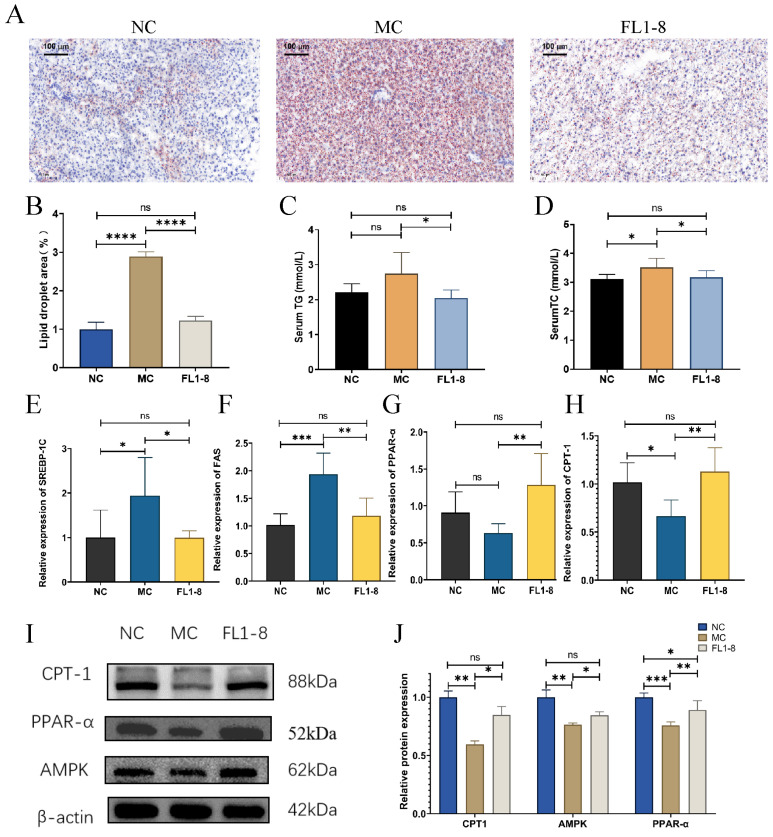
The effect of lipid metabolism in model mice after treatment by *L. rhamnosus* NKU FL1-8. (**A**) Liver oil red O staining; (**B**) lipid droplet area; (**C**) serum TG levels; (**D**) serum TC level; (**E**) hepatic SREBP-1C gene expression; (**F**) hepatic FAS gene expression level; (**G**) PPAR-α gene expression level in liver; (**H**) hepatic CPT-1 gene expression level; (**I**) hepatic CPT1, PPAR-α, and AMPK protein expression; (**J**) protein expression of CPT1, PPAR-α and AMPK in liver. Data are mean ± standard deviation (*n* = 8); * *p* < 0.05**, *p* < 0.01, *** *p* < 0.001, **** *p* < 0.0001, ns: *p* > 0.05.

As shown in Figure 4A, the MC group had a larger volume and a greater number of liver fat droplets than the NC group, and the FL1-8 group had a significantly smaller size and number of lipid droplets than the mice in the MC group, indicating that the intervention of *L. rhamnosus* NKU FL1-8 significantly reduced the level of liver fat vacuolization caused by gavage of alcohol (Figure 4B). In terms of TG and TC levels, the MC group was significantly higher than the NC group, and the FL1-8 group was significantly lower than the MC group (Figure 4C,D). These results suggest that *L. rhamnosus* NKU FL1-8 intervention can effectively reduce the formation of alcoholic fatty liver induced by alcohol intake in mice. Alcoholic fatty liver is caused by the host’s excessive alcohol consumption, resulting in the production of acetaldehyde, reactive oxygen species, and endoplasmic reticulum stress. Then, the fatty acid oxidation of adenosine 5′-monophosphate (AMP)-activated protein kinase (AMPK) and carnitine palmitoyltransferase 1 (CPT-1) was blocked [18]. Therefore, analysis of the changes in AMPK and CPT-1 at the protein level is an important parameter to evaluate the host fatty acid oxidation level. As shown in Figure 4I,J, the MC group’s protein expression level of CPT-1 and AMPK has a downward trend, while the FL1-8 group can increase the expression of these proteins. The changes in CPT-1 at the gene level were also the same as those at the protein level (Figure 4H–J).

In addition, alcohol consumption induces up-regulation of sterol regulatory element binding protein 1c (SREBP-1c) levels, which are responsible for fatty acid synthesis through fatty acid synthase (FAS) [19]. “Hepatic SREBP1c and FAS expression levels were up-regulated in the livers of alcohol-treated mice but were significantly reduced in mice treated with *L. rhamnosus* NKU FL1-8 (Figure 3E,F)”. “Alcohol-treated mice showed significant reductions in both gene and protein expression of PPAR-α in the liver (Figure 4G,I,J), which may lead to a reduction in fatty acid β-oxidation. The treatment *L. rhamnosus* NKU FL1-8 significantly reversed the alcohol exposure-induced changes in PPAR-α levels, which may contribute to the reduction in fatty acid synthesis”.

### 3.5. Intestinal Barrier Integrity

**Figure 5:**
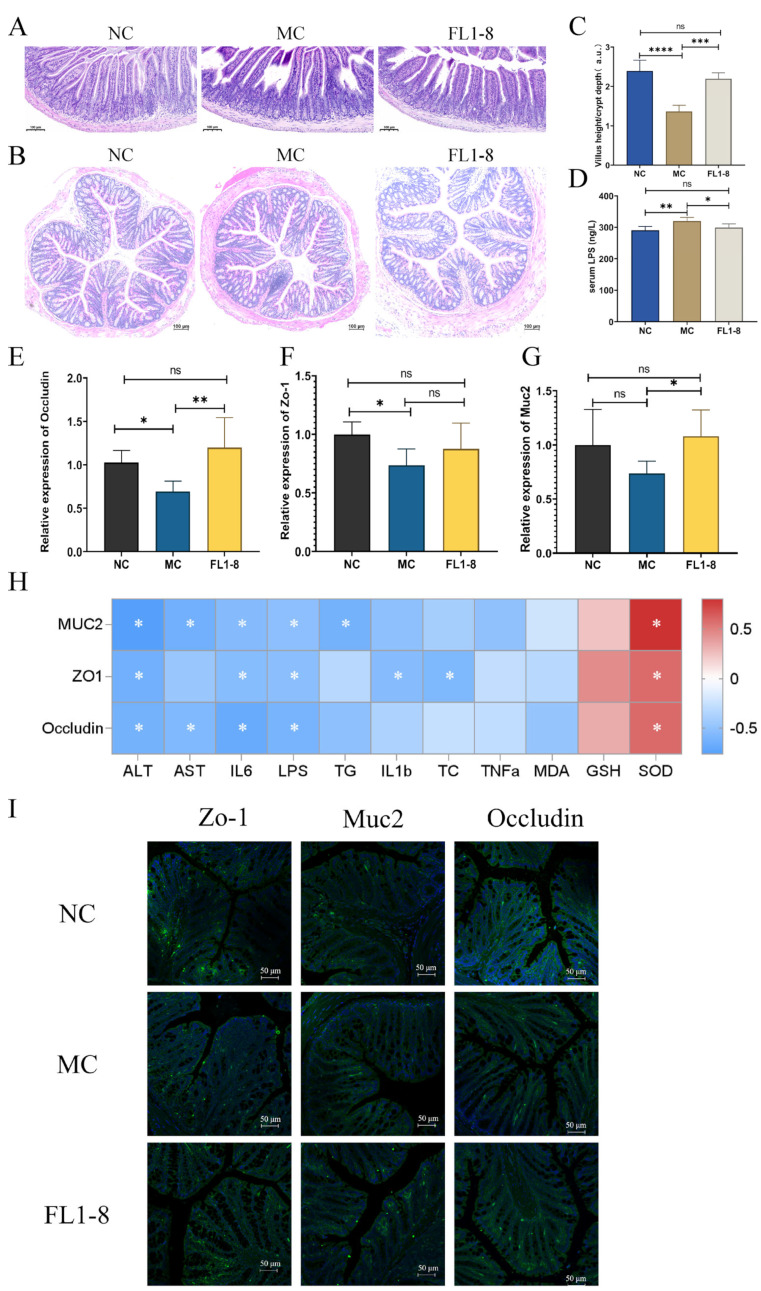
Effect of *L. rhamnosus* NKU FL1-8 supplementation on alcohol-induced intestinal barrier in mice. (**A**) H&E staining of ileum; (**B**) the colon HE staining; (**C**) villus length/crypt depth ratio; (**D**) serum LPS level; (**E**–**G**) mRNA expression of occludin, Zo-1 and Muc2 genes in colon; (**H**) correlation analysis of colonic occludin, Zo-1, and Muc2 mRNA expression levels with other factors (**I**) colonic occludin, ZO-1 and MUC2 protein expression levels (green fluorescence indicates tight junction proteins, and blue fluorescence indicates DAPI). Data are mean ± standard deviation (*n* = 8); * *p* < 0.05, ** *p* < 0.01, *** *p* < 0.001, **** *p* < 0.0001, ns: *p* > 0.05.

Alcohol and its metabolite, acetaldehyde, increased intestinal permeability, releasing its harmful lipopolysaccharide (LPS) and other substances into the blood stream [16]. As shown in Figure 5A,C, necrotic loss of epithelial cells and sparse arrangement of villi were observed in the ileal mucosa of the MC group. In contrast, it showed a more regular morphology and longer villi in the FL1-8 group, and significant changes in villus length in the small intestine were also observed. In addition, compared with the NC group, the colon tissues of the MC group showed local inflammation with an irregular arrangement of villi, and the colonic H&E staining of the FL1-8 group showed alleviation of inflammation (Figure 5B). In terms of intestinal barrier-related genes, the mRNA expression levels of Zo-1 and Occludin were significantly decreased, but Muc2 was not significantly changed in alcohol-treated mice. *L. rhamnosus* NKU FL1-8 ameliorated the alcohol-induced decrease in tight junction protein mRNA levels. Interestingly, Muc2 mRNA expression was also significantly increased in the FL1-8 group compared with the NC group (Figure 5E–G).

Adding mice to *L. rhamnosus* NKU FL1-8 increased Zo-1, Muc2, and occludin protein levels (Figure 5I). These results are consistent with the qPCR detection of intestinal gene expression levels. When the intestinal barrier is breached, LPS is released from the intestine into the blood. Bacterial LPS is the main outer mask component of most Gram-negative bacteria. When it is excessive, it will cause local and systemic inflammation, thus causing a certain degree of damage to the liver [19]. We therefore also examined LPS levels in the blood. Compared with the NC group, the MC group had a significant increase in blood LPS levels, while the FL1-8 group had a significant reduction in LPS levels compared with the MC group and no significant change compared with the NC group (Figure 5D). In addition, we also performed correlation analyses for these factors. As shown in Figure 5H, colon occludin expression level was negatively correlated with liver AST, ALT, IL-6, LPS, and TG levels. The expression of Zo-1 in the colon was negatively correlated with the levels of ALT, IL-6, IL-1β, LPS, and TC in the liver. Colon Muc2 expression was negatively correlated with liver AST, ALT, IL-6, and LPS levels. The expression levels of three typical intestinal barrier function genes were positively correlated with liver SOD levels. These results suggest that *L. rhamnosus* NKU FL1-8 treatment has a strong protective effect on the intestinal barrier, and the increased level of intestinal barrier function is likely to be an important mechanism for the reduction of host inflammation and the increase of antioxidant capacity, thereby protecting the host liver.

### 3.6. Gut Microbiota Composition

**Figure 6:**
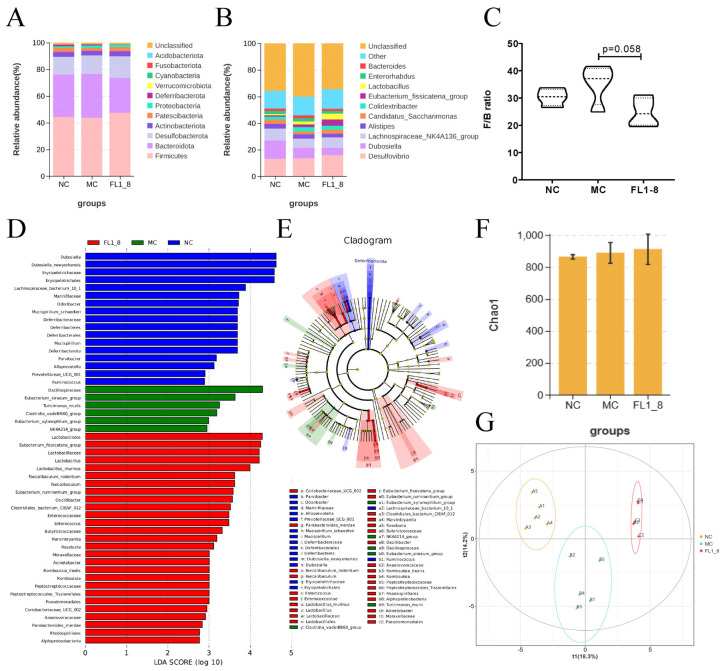
Effect of *L. rhamnosus* NKU FL1-8 supplementation on alcohol-induced gut microbiota in mice. (**A**) Gate stack level species distribution diagram; (**B**) stacked map of species distribution at genus level; (**C**) the ratio of Firmicutes to Bacteroidota; (**D**) LEfSe bar graph; (**E**) LEfSe ring graph; (**F**) Chao index analysis; (**G**) PLS-DA analysis. Data are presented as mean ± standard deviation (*n* = 5).

From the perspective of species richness, at the phylum level (Figure 6A), the Proteobacteria phylum abundance was significantly increased in the MC group compared with the NC group. The abundance of Deferribacterota was decreased, and the ratio of Firmicutes to Bacteroidota was increased in the FL1-8 group as compared with the MC group (Figure 6C). The ratio of Firmicutes to Bacteroidetes is an important index to measure the balance of the gut microbiota.

At the genus level, the MC group significantly increased the abundance of *Eubacterium_ruminantium_group*, *GCA-900066575* and *NK4A214_group* compared with the NC group. Significantly reduced the abundance of *Parvibacter* and *Prevotellaceae_UCG-001*. Compared with the MC group, the abundance of *Mucispirillum*, *Odoribacter*, and *NK4A214_group* was significantly reduced in the FL1-8 group, and significantly increased the *Faecalibaculum* and *Coriobacteriaceae_UCG-002 genera* (Figure 6B). LEfSe analysis was used to identify genus abundance. In the MC group, *Eubacterium_xylanophilum_group* and *Clostridia_vadinBB60_group* increased compared with the other two groups. *Lactobacillus*, *Faecalibaculum*, *Eubacterium_ruminantium_group*, *Romboutsia*, *Oscillibacter,* and so on were significantly increased in the FL1-8 group compared with the MC and NC groups. Beneficial and neutral bacteria (Figure 6D,E). As shown by the Chao index analysis, there was no significant difference in diversity among the three groups (Figure 6F). By partial least squares-discriminate analysis (PLS-DA), it was found that the flora was quite different among the three groups, and there was an obvious clustering phenomenon (Figure 6G). These results suggest that alcohol intervention has a great effect on the composition of the gut microbiota, and *L. rhamnosus* NKU FL1-8 can restore the gut microbiota disorder caused by acute alcohol intake by increasing the abundance of beneficial bacteria and reducing the relative abundance of harmful bacteria.

## 4. Discussion

Alcohol intake affects liver health in a number of ways, including abnormal liver function, oxidative damage, and inflammation. It may also affect the gut microbiota, leading to liver damage through the gut–liver axis [20]. Probiotics regulate gut microbial homeostasis and treat a variety of liver diseases, including nonalcoholic fatty liver disease, cirrhosis, and ALD [21]. Preclinical studies have reported that probiotic pretreatment can prevent ALD by remodeling the gut microbiota and reducing alcohol-induced infusions of LPS and fat [22]. Studies have shown that planllular products of alcohol-induced liver and intestinal injury are protective [23].

*L. rhamnosus* NKU FL1-8 was effective in alleviating liver damage induced by acute alcohol intake in mice. *L. rhamnosus* NKU FL1-8 reduced ALT levels, increased antioxidant capacity, and reduced liver and colonic inflammation in the host. In addition, *L. rhamnosus* NKU FL1-8 can enhance the intestinal barrier function and reduce the toxicity of LPS to the host by up-regulating the expression of tight junction mucin, thereby reducing systemic inflammation [24]. *L. rhamnosus* NKU FL1-8 most likely remodels the microbiota to ameliorate alcohol-induced gut microbiota dysbiosis and impaired gut barrier function, thereby protecting the liver. AST and ALT activities are the most sensitive indicators of liver function, which can effectively reflect the degree of liver cell damage. Acute alcohol intake causes liver toxicity, which leads to increased serum ALT and AST levels [25]. When hepatocytes are damaged by various external factors, the following changes occur: cell degeneration, necrosis, and increased permeability of the cell membrane. Alcohol metabolism leads to increased cell membrane permeability and mitochondrial damage [14], which in turn leads to the release of alanine aminotransferase and aspartate aminotransferase from mitochondria and cytoplasm into the blood, thereby increasing the activity of alanine aminotransferase and alanine aminotransferase in serum [26]. *L. rhamnosus* NKU FL1-8 could significantly reverse the levels of ALT and AST. It has been reported in the literature that the serum AST and ALT activities after intervention were lower than those of the gavage alcohol group, suggesting that the intervention has a potential role in protecting and repairing alcohol-induced liver damage [27]. Ethanol stimulation can cause abnormal expression of inflammatory factors (IL-6, IL-1β, and TNF-α) in serum. TNF-α causes damage to hepatocytes and induces the production of IL-1β and IL-6, thereby causing hepatocyte apoptosis and liver inflammation [28]. 

In our research, ethanol stimulation also increased IL-6, IL-1β, and TNF-α levels in mice, while *L. rhamnosus* NKU FL1-8 intervention significantly reduced these levels. Clinically, acute alcohol intake often leads to systemic, low-grade inflammation [29]. In addition, alcohol-induced oxidative stress accelerates the oxidation of polyunsaturated fatty acids, resulting in MDA production. As the final product of lipid peroxidation, MDA content in the body is often used to characterize the degree of oxidative stress in the body. At the same time, the liver’s glutathione antioxidant system is considered to be the key factor in alcoholic liver damage [30]. Our results showed that alcohol exposure caused a significant decrease in GSH and SOD levels and an increase in MDA levels, indicating a decrease in host liver antioxidant capacity and an accumulation of polyunsaturated fatty acid oxidation products. In Ruofan Liu’s study, it was found that acute alcoholic liver damage can reduce the level of SOD in the host, and the reduction in SOD activity indicates a certain degree of oxidative stress in the liver [31]. SOD activity was significantly increased after *L. rhamnosus* NKU FL1-8 gavage, indicating an enhanced ability of hepatocytes to scavenger oxygen-free radicals and inhibit oxidative stress. Previous studies have also pointed out that the improvement of the hepatic GSH antioxidant system can improve alcohol-induced liver damage [32]. Similarly, in Xiaowei Xu’s study, *Lactiplantibacillus plantarum* P101 was used to alleviate alcoholic fatty liver disease, and a reversal of MDA and SOD levels was also found in the probiotic group. This may be related to the fact that *L. rhamnosus* regulates the gut microbiota of the host, thereby increasing the levels of metabolites of antioxidant function in the gut. Subsequently, these substances enter the enterohepatic axis circulation through the portal vein, thereby reducing liver and even systemic oxidative stress damage. In addition, our study also found that the expression of Nrf2, HO-1, and NQO1 genes was up-regulated in the MC group, which was alleviated by our intervention. When oxidative stress occurs in vivo, the degradation of Nrf2 is interrupted, regulating the levels of a range of antioxidant genes to resist oxidative damage in vivo. Activation of Nrf2 can alleviate many types of liver diseases, including acute inflammatory liver damage, chemical liver damage, alcoholic liver damage, and nonalcoholic steatohepatitis [33]. Alcohol-induced signaling cascades are generally thought to be initiated through the Nrf2/HO-1 pathway, suggesting that HO-1 may be a potential target for the interplay of inflammation and oxidative stress in ALD [34]. Enhanced HO-1 expression is an endogenous defense mechanism used by cells to mitigate injury. Therefore, HO-1 production is prevalent in various liver injuries. Consistent with the results of Huang et al., the increased expression of HO-1 may represent an improvement in liver damage [35]. These results suggest that *L. rhamnosus* may regulate the host oxidative stress system through the Nrf2 pathway, which is likely to be the key mechanism by which *L. rhamnosus* NKU FL1-8 protects the host against alcoholic liver damage. In brief, *L. rhamnosus* NKU FL1-8 intervention significantly ameliorated acute alcoholic liver damage by inhibiting oxidative damage and modulating inflammatory pathways. SREBP-1c is a transcription factor that can activate the fatty acid synthesis of all the genes [18].

Alcohol exposure significantly increased liver lipid accumulation, liver damage, and hepatocyte apoptosis in mice, and *L. rhamnosus* NKU FL1-8 effectively prevented these changes. The protective effect of *L. rhamnosus* NKU FL1-8 on fat accumulation may be related to metabolism, including *de novo* lipogenesis and catabolism. Hepatic SREBP-1c gene expression levels were upregulated in alcohol-treated mice during the development of hepatic steatosis. This phenomenon was also reported similarly in Jing Tian’s article [36]. The mechanism of alcohol-related steatosis is thought to be the production of acetaldehyde, reactive oxygen species, and ER stress resulting in steatosis due to chronic alcohol consumption, with the result that AMPK is blocked. CPT1 is a key enzyme in carnitine-dependent transport across the inner mitochondrial membrane, and the function of AMPK responsible for fatty acid oxidation is mediated through CPT-1 [18,37,38]. PPAR-α is a transcription factor and a major regulator of hepatic lipid metabolism. Activation of PPAR-α promotes fatty acid uptake, utilization, and catabolism by up-regulating genes involved in peroxisomal and mitochondrial fatty acid β-oxidation [39]. In the liver tissue of mice treated with alcohol, the expression of the PPAR-α gene and fatty acid β-oxidation was decreased significantly, thus the drops in the accumulation of hepatic fat were increased. The expression of CPT-1 can be significantly promoted by up-regulating the expression of PPAR-α [40]. Intervention with *L. rhamnosus* NKU FL1-8 significantly reversed the alcohol exposure-induced changes in these genes. We hypothesized that *L. rhamnosus* NKU FL1-8 could inhibit the formation of fatty liver by increasing the expression of AMPK and PPAR-α, thereby promoting the expression of CPT-1. The integrity of the intestinal barrier ensures the normal transport of nutrients, electrolytes, and water, while acting as a protective barrier to prevent the displacement of luminal toxins and gut microbiota [41]. Previous studies have reported disarrangement of epithelial cells, reduced crypt depth, and impaired intestinal architecture in alcohol-exposed mice [42]. Our findings are in good agreement with these observations. Tight junction proteins are major components of cell–cell adhesion complexes in the intestinal barrier and play a key role in controlling paracellular permeability and improving epithelial barrier integrity. Tight junction proteins, including occludin and Zo-1, are important markers of the intestinal barrier and are becoming targets for new therapeutic approaches to liver disease. As a transmembrane integration protein, occludin mainly contributes to the establishment of cell–cell tight junctions and barrier function. The zonula occludens-1 (Zo-1) level is used as a biomarker of intestinal barrier dysfunction [43]. Our findings showed that alcohol intake caused a significant decrease in the expression of tight junction proteins and increased the amount of LPS in the blood, indicating that intestinal integrity was already severely compromised. *L. rhamnosus* NKU FL1-8 could restore the reduced tight junction protein levels, including Zo-1 and occludin protein immunofluorescence results and qPCR detection of gene expression levels. In addition, although we did not observe a reduction in the expression of Muc2 in colon tissues, Muc2 is an important mucus protein secreted by goblet cells in the colon [44], which plays an important role in the integrity of the intestinal barrier, and the intervention of *L. rhamnosus* NKU FL1-8 can significantly increase the protein level of Muc2. This suggests that it has a unique effect on intestinal barrier function. These results suggest that *L. rhamnosus* NKU FL1-8 can reduce ethanol-induced intestinal damage, especially through the destruction of the intestinal barrier, which could help alleviate the damage caused by alcohol to the liver, [45]. The gut microbiota is a complex and dynamic ecosystem that is essential for maintaining the integrity of the intestinal barrier and intestinal homeostasis [46]. In recent years, a large number of studies have shown that gut microbiota is an important factor mediating the gut–liver axis [47]. As a diol, alcohol itself has a strong inhibitory effect on the growth of microorganisms, and its large intake may lead to microbial ecological imbalance, especially the regulation of the growth and metabolism of some probiotics and functional neutral bacteria, which may be an important factor in alcohol-induced liver damage [48].

Acute alcohol intake led to gut microbial dysbiosis, particularly changes in diversity between groups. It has been shown [49] that alcohol exposure decreases the relative abundance of Firmicutes, increases the relative abundance of Bacteroidetes, and increases the Firmicutes/Bacteroidetes ratio, which is consistent with our findings. Lavage after alcohol can increase the abundance of harmful bacteria in the gut, and *L. rhamnosus* NKU FL1-8 intake can resist the change and change the gut microbial diversity, whose character is raised after gastric *L. rhamnosus* NKU FL1-8 *Lactobacillus* abundance: we suspect this may be due to the lavage of *L. rhamnosus* NKU FL1-8. *L. rhamnosus* increased markedly in the body and in engraftment. Our results showed that *L. rhamnosus* NKU FL1-8 increased *Faecalibaculum*, *Eubacterium_ruminantium_group*, *Romboutsia*, and *Oscillibacter*. Oscillibacter has been reported to regulate lipid biosynthesis [50]. In brief, our research confirms some previous findings that probiotic supplementation can ameliorate alcohol-induced liver damage. Probiotics, including *Lactobacillus rhamnosus* GG, have been shown to suppress inflammatory signals in experimental colitis [51]. Probiotics can also directly modulate immune cell functions, including dendritic cells, macrophages, and natural killer T cells. A recent study by Forsyth et al. showed that probiotics can activate NF-κB, which has been identified as a key pathway in alcohol-induced oxidative stress and intestinal hyperpermeability [51].

Future research should aim to further reveal the correlation between gut microbiota and alcohol abuse-induced host physiological function damage, especially liver function damage, as well as its potential mechanism of action, and try to prevent the disorder of gut microbiota or find key microorganisms to reverse this damage, so as to serve as a new therapeutic target. Although we have not yet used this strain for intervention in a clinical setting, the present results give us considerable confidence.

## 5. Conclusions

**Figure 7:**
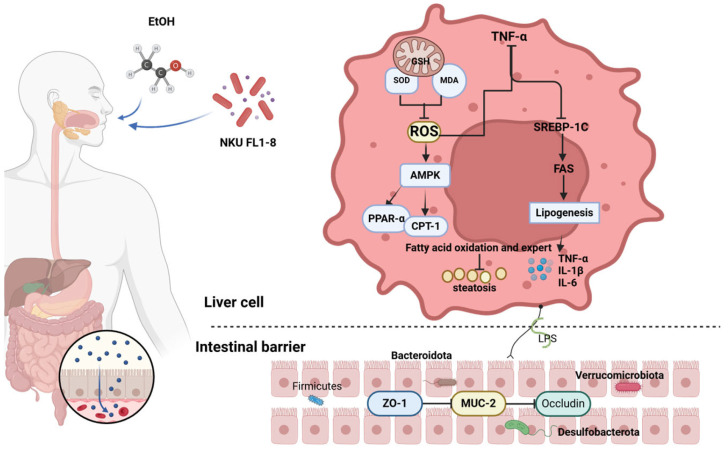
The mechanism of *L. rhamnosus* NKU FL1-8 ameliorates alcoholic liver damage (Created with BioRender.com).

To sum up, treatment orally via *L. rhamnosus* NKU FL1-8 isolated from infant feces could relieve acute alcoholic liver damage in mice. *L. rhamnosus* NKU FL1-8 alleviates acute alcoholic liver damage by improving the liver SOD antioxidant system, reducing liver and colon inflammation levels, maintaining intestinal epithelial integrity, and regulating intestinal microbiota homeostasis. We hypothesize that *L. rhamnosus* NKU FL1-8 regulated the gut microbiota and repaired the intestinal barrier to reduce the level of LPS in the blood, thereby reducing oxidative stress and systemic inflammation. In conclusion, our data suggested that *L. rhamnosus* NKU FL1-8, as a safe probiotic strain with the potential to alleviate acute alcoholic liver damage, could be used as a potential functional food for the prevention of ALD (Figure 7).

## Figures and Tables

**Table 1:** Genes and primers selected for qPCR.

Gene	Primer	Sequence (5′ to 3′)
*β-actin*	Forward	ACAGCAGTTGGTTGGAGCAA
	Reverse	ACGCGACCATCCTCCTCTTA
*Nrf2*	Forward	CTTTAGTCAGCGACAGAAGGAC
	Reverse	AGGCATCTTGTTTGGGAATGTG
*HO-1*	Forward	AGGGCAGAAGGGAATTGCTC
	Reverse	AAAGAGCTGGAGAGCCAACC
*NQO-1*	Forward	GAACCCAGTCTATGCCCCAC
	Reverse	GGCGTGCAAGGGATGATTTC
*PPARα*	Forward	TTTCAAGGGTGCCAGTTTCG
	Reverse	CCATCTTTATTCATCAGGGAGGC
*Srebp-1c*	Forward	GGAGCCATGGATTGCACATT
	Reverse	GGCCCGGGAAGTCACTGT
*FAS*	Forward	TGCTCCCAGCTGCAGGC
	Reverse	GCCCGGTAGCTCTGGGTGTA
*CPT-1*	Forward	GTGACTGGTGGGAGGAATAC
	Reverse	GAGCATCTCCATGGCGTAG
*Zo-1*	Forward	GCCGCTAAGAGCACAGCAA
	Reverse	GCCCTCCTTTTAACACATCAGA
*Muc2*	Forward	ATGCCCACCTCCTCAAAGAC
	Reverse	GTAGTTTCCGTTGGAACAGTGAA
*Occludin*	Forward	GCGGAAGAGGTTGACAGTCC
	Reverse	AGGAGCGAGACCCCACTAA
*16s V3-V4*	Forward	CCTACGGGNGGCWGCAG
	Reverse	GGACTACHVGGGTATTCTAAT

## Data Availability

The original contributions presented in the study are included in the article, further inquiries can be directed to the corresponding author.
